# A rapid-crosslinking antimicrobial hydrogel with enhanced antibacterial capabilities for improving wound healing

**DOI:** 10.3389/fphys.2023.1206211

**Published:** 2023-05-30

**Authors:** Xi Zhang, Wanxin Li, Genying Wei, Yuling Yan, Ruitao He, Yan Wang, Daoyuan Chen, Xiaofei Qin

**Affiliations:** ^1^ School of Biological Engineering, Zunyi Medical University, Zhuhai, Guangdong, China; ^2^ Department of Clinical Medicine, The Fifth Clinical Institution, Zhuhai Campus of Zunyi Medical University, Zhuhai, Guangdong, China

**Keywords:** hydrogel, rapid-crosslinking, antimicrobial, anti-inflammatory, wound healing

## Abstract

One of the main reasons impeding wound healing is wound infection caused by bacterial colonization with a continuous stage of inflammation. Traditional wound treatments like gauze are being replaced by tissue adhesives with strong wet tissue adhesion and biocompatibility. Herein, a fast-crosslinking hydrogel is developed to achieve both strong antimicrobial properties and excellent biocompatibility. In this study, a simple and non-toxic composite hydrogel was prepared by the Schiff base reaction between the aldehyde group of 2,3,4-trihydroxybenzaldehyde (TBA) and the amino group of *ε*-Poly-_*L*_-lysine (EPL). Subsequently, a succession of experiments toward this new hydrogel including structure characterization, antimicrobial properties, cell experiment and wound healing were applied. The results of the experiments show that the EPL-TBA hydrogel not only exhibited excellent contact-active antimicrobial activities against Gram-negative bacteria *Escherichia coli* (*E. coil*) and Gram-positive Bacteria *Staphylococcus aureus* (*S. aureus*), but also inhibited the biofilm formation. More importantly, the EPL-TBA hydrogel promoted the wound healing with low cytotoxicity *in vivo*. These findings indicate that the EPL-TBA hydrogel has a promising use as a wound dressing in the bacterial infection prevention and wounds healing acceleration.

## 1 Introduction

The skin is located on the surface of the body and has the function of sensing the outside world and preventing invasion of the body by external bacteria and pathogens (Hoque and Haldar, 2017). The occurrence of wounds is often incidental, and the usual healing time will be 8–12 days (Addor et al., 2012). Once the skin is damaged, exposed tissues will bleed and be colonized by germs, delaying tissue repair (Diegelmann and Evans, 2004; Addor, et al., 2012). If the wound infection is not treated in a timely manner, the resulting complications will be life-threatening to the patient’s life. Certain currently utilized wound dressing materials, such as gauze and tissue adhesives, have disadvantages such as poor antibacterial properties, poor mechanical performance, and inability to deliver moisture to accelerate wound healing (Alven and Aderibigbe, 2020). In contrast, hydrogel is considered an ideal material for wound treatment because of its excellent moisturizing properties, water absorption, and antibacterial property (Asadi et al., 2021; Yu et al., 2022).

Currently, hydrogel, the hydrophilic polymers with three-dimensional reticular structure come into view, have both liquid and solid properties (Jeong et al., 2002; Zhang and Khademhosseini, 2017; Caccavo et al., 2018; Zhang et al., 2020). Due to its degradability and excellent biocompatibility, hydrogels have been widely used in wound infection, drug control and release, artificial blood and skin, and flexible electronics (Zhang, et al., 2020). This category of biomaterials combines multiple advantages for wound repair, including biocompatibility, degradability, tunable mechanical properties, high water content, strong tissue adhesion, outstanding antibacterial activity, and excellent substrates for drug delivery (Ghobril and Grinstaff, 2015). Therefore, it is now currently considered the most effective material for solving wound infection (Zhai et al., 2017; Fan et al., 2019; Nikjoo et al., 2021). So far, hydrogels have been developed by chemical cross-linking, physical cross-linking and other methods (Nikjoo, et al., 2021). Special chemical alterations of synthetic materials and the employment of special chemical cross-linking agents are frequently required during the creation of hydrogels, resulting in slow cross-linking times that greatly limit the application of hydrogels (Yuan et al., 2021). Therefore, it is essential to produce hydrogel dressings with simple processes, fast cross-linking time, acceptable physical and chemical properties, and excellent biocompatibility using natural polymers.

Antimicrobial peptides (AMPs) are a class of small-molecule peptides with functions against external pathogens (Jenssen et al., 2006). AMPs are widely found in a variety of organisms and are an important part of the nonspecific immune function of organisms, with various biological functions such as anti-bacterial, viral, fungal, and tumor activities (Jenssen, et al., 2006; Caccavo, et al., 2018). Furthermore, the special bactericidal mechanism of antimicrobial peptides makes bacteria less susceptible to drug resistance, showing excellent application prospects in several fields and promising to be a new type of green antimicrobial molecule or antimicrobial additive (Yan et al., 2021; Mehta et al., 2022).

*ε*-Poly-_*L*_-lysine (EPL) is a natural-based peptide with antibacterial properties, and this biological preservative was first used in food preservation in the 1980s. EPL can be broken down in the human body to lysine, which is one of the eight essential amino acids and is allowed to be fortified in food products worldwide [26]. Moreover, EPL has been approved and allowed as a food additive in Japan, Korea, and the United States, and studies have been conducted to confirm that EPL does not cause mutations in bacteria and other pathogens (Shima et al., 1984). Due to the amino group-containing chain which could grafted with other active molecules or polymers, hydrogels can be designed from EPL with excellent biocompatibility and adhesion properties (Dong et al., 2021). Previously, Xu *et al.* reported the preparation of hydrogels using EPL with catechol (CT) through Schiff base reaction, and the hydrogel showed promising antibacterial activities (Xu et al., 2019). reported a hydrogel formed using methacrylic acid and EPL by chemical cross-linking that exhibited broad-spectrum antibacterial activity (Zhou et al., 2011). However, these hydrogels were formed using complex synthesis process, resulting in a long formation time. The complex method of synthesis and slow cross-linking rate greatly limit the commercial applications of hydrogels.

2,3,4-trihydroxybenzaldehyde (TBA) has received some attention due to its antimicrobial properties (Zhou et al., 2023). Moreover, TBA can form a series of one-pot physically synthesized hydrogels with various polymers because of the containing phenolic hydroxyl and aldehyde groups (He et al., 2021).

Herein, we designed a fast cross-linked hydrogel (EPL-TBA) by the Schiff base reaction between EPL and TBA at pH equal to 8.5. In our study, the rapid cross-linking hydrogel were formed within only 5 min. In our study, the physicochemical characteristics like swelling and moisturizing properties of EPL-TBA hydrogels were tested. Antibacterial tests were also conducted on the hydrogel, and showing broad-spectrum antibacterial ability. For biosafety issues, cytotoxicity and hemolysis tests were performed to confirm the excellent biocompatibility of hydrogels. Finally, a wound model using rats was used to demonstrate that EPL-TBA hydrogel aids in the healing process of wounds. Our designed hydrogel can be cross-linked formed in as little as 5 min, and it possesses both excellent antibacterial properties and good biocompatibility, providing a new idea and method for solving wound healing problems in the future.

## 2 Materials and methods

### 2.1 Materials

*ε*-Poly-_*L*_-lysine (EPL) (consisting of 25–30 _L_-lysine residues) was purchased from Zhengzhou Bainafo Bioengineering Co. Ltd. (China). 2,3,4-trihydroxybenzaldehyde, sodium hydroxide, hydrogen peroxide (30%) and Tris**-**HCl buffer were all purchased from Shanghai Maclin Reagent Co., LTD. *Staphylococcus aureus* (*S. aureus*), *Escherichia coli* (*E. coli*), methicillin-resistant *Staphylococcus aureus* (*MRSA*) and *Pseudomonas aeruginosa* (*P. aeruginosa*) were derived from The Fifth Affiliated Hospital of Zunyi Medical University. Human skin fibroblast (BNCC337722) were derived from the cell sharing platform of Zhuhai Campus of Zunyi Medical University. DMEM culture medium and fetal bovine serum were derived from Kgi Reagent CD, LTD. NO kit is from Nanjing Jiancheng Bioengineering Institute. IL-6 and TNF-α kits were derived from Jiangsu Enzyme Free Industrial Co., LTD.

### 2.2 Preparation of EPL-TBA hydrogel

The hydrogels were created in a single step using a modified version of a previously reported method (Xu, et al., 2019). In short, TBA (247 mM, 371 mM and 494 mM) in Tris-HCl buffer (pH = 8.5, 1 M) in 25°C dissolves oscillations. Then add 1 M sodium hydroxide and EPL and shake to dissolve it. Finally, add 50 μL H_2_O_2_ (30 wt%) in glass bottles and incubate at room temperature for 5min to form a hydrogel. The hydrogels with different concentrations were named EPL-TBA-40, EPL-TBA-60 and EPL-TBA-80. The 40, 60 and 80 in EPL-TBA-40, EPL-TBA-60 and EPL-TBA-80 refer to the quality concentration of TBA (mg/mL), which equal to the molar concentration 247mM, 371mM and 494 mM of EPL-TBA-40, EPL-TBA-60 and EPL-TBA-80, respectively.

### 2.3 Characterization of the EPL-TBA hydrogel

#### 2.3.1 FT-IR and UV-vis spectrophotometer analysis

To confirm the interaction between EPL and TBA a Fourier transform infrared spectrometer (FT-IR Shimadzu Company of Japan) was employed to analyze the EPL-TBA hydrogels. The FT-IR spectra were captured at 25 °C in the 4,000–400 cm^−1^ wavenumber range. A UV-vis spectrophotometer (Agilent Cary 3,500) was used to measure the UV-vis spectra of the hydrogels EPL, EPL-TBA-40, EPL-TBA-60 and EPL-TBA-80.

#### 2.3.2 Swelling and moisture content studies

The swelling capacity of different EPL-TBA hydrogels was evaluated by measuring the weight changes before and after the hydrogels in PBS solution (Chen et al., 2021). Briefly, freeze-dried hydrogels (*W*_*0*_) were placed in PBS solutions at different temperatures and removed after a period of time. The weight of the hydrogel (*W*_*1*_) is measured after absorbing the remaining liquid on the surface of the hydrogel with filter paper. The percentage of swelling was then calculated using the Eq.

$$Swelling\left(\%\right)=\frac{W_{1}-W_{0}}{W_{0}}\times 100\%$$

To measure the water retention ratio of EPL-TBA hydrogels, after formation of the hydrogels, the weights of the hydrogels (*W*_*0*_) were determined. Then they were separately placed in the incubator at 37°C. At predetermined time points, the weight of the hydrogel was measured (*W*_*1*_). The percentage of Water holding ratio was calculated using the Eq.

$$Water\;holding\;ratio\left(\%\right)=\frac{W_{1}}{W_{0}}\times 100\%$$

### 2.4 Rheological behavior of EPL-TBA hydrogels

EPL-TBA hydrogels were recorded at 37°C on a rheometer (MCR302, Anton Paar, Austria). The rheometer is equipped with parallel plates with a diameter of 25 mm. The frequency scanning range was 0.1–15 Hz, the temperature was set at 37°C, and the constant strain was 1% to test the stability of EPL-TBA hydrogel.

### 2.5 Minimum inhibitory concentration (MIC) assay

The antibacterial activities of EPL and TBA against *S. aureus* and *E. coli* were determined by MIC method (Chen et al., 2019). Briefly, the chosen bacteria were able to grow in LB medium until the mid log phase. Dilute the bacterial working suspension to 10^6^ (CFU/mL). On a 96-well plate, 50 μL of EPL/TBA in LB (4,000 μg/mL) was 2-fold diluted and then mixed with 50 μL of a bacterial culture containing 10^6^ CFU/mL. The final bacterial concentration was 1 × 10^5^ CFU/mL. The OD value of bacteria was determined at OD600_nm_. If no bacterial growth is observed, this concentration is the MIC value of the bacteria.

### 2.6 Antimicrobial activity of EPL-TBA hydrogel

The Gram-positive bacteria *S. aureus* and *MRSA* and Gram-negative bacteria *E. coli* and *P. aeruginosa* were employed to assess the antibacterial efficacy of the EPL-TBA hydrogel (Zhou et al., 2022). Briefly, 1 mL of hydrogel was put into a clean glass bottle, and then 400 μL of bacteria in the mid-log phase in LB medium were diluted to 1 × 10^5^ CFU/mL and added to each bottle, which was then incubated at 37°C for 24 h. At OD_600 nm_, the absorbance of bacteria at different time points was measured. Moreover, the bacterial suspension was daubed onto the agar plate, and the dish was cultured for an additional 24 h.

### 2.7 Morphological observation of bacteria

The morphology of bacteria treated with EPL-TBA hydrogel was observed using SEM. Briefly, The EPL-TBA hydrogel was incubated with bacteria for 4 h and fixed with 2.5% glutaraldehyde. The samples were freeze-dried by freeze-drying machine and observed by SEM after 24 h of freeze-drying.

### 2.8 Biological film assay

The concentration of Gram-positive bacteria *MRSA* was diluted to 1 × 10^8^ CFU/mL, which was used as the bacterial working bacterial suspension (Zhi et al., 2017). Incubate the EPL-TBA hydrogel and quartz plate in a working bacterial suspension for 1 day to allow the biofilm to multiply. The surface of the EPL-TBA hydrogel and quartz plate was rinsed with PBS buffer to remove suspended bacteria. The hydrogel and quartz plates were then dyed with the LIVE/DEAD Backlight kit. The production of biofilm was observed under confocal microscope. Finally, the software was used for photography as well as data analysis.

### 2.9 Cytotoxicity determination

Before being used for 3 days, the EPL-TBA hydrogel was made in a Petri dish, ground into a powder with a grinder, and disinfected with PBS. Around 5,000 human skin fibroblasts (BNCC337722) were plated per well in 96-well plates. In DMEM with 10% (v/v) fetal bovine serum, cells were grown (FBS). At 37°C, cells were grown in a humidified environment with 5% CO_2_. The cytotoxicity of the EPL-TBA hydrogel toward human skin fibroblast (BNCC337722) cells was determined by CCK8. The percentage of cell survival was calculated using the Eq. After incubation with a hydrogel soak, BNCC337722 cells were stained with a LIVE/DEAD staining kit to evaluate the cytocompatibility of BNCC337722 cells. Incubate and stain at room temperature for 30 min, thoroughly clean the samples with PBS, and take photos of the staining results with an inverted microscope.

$$Cell\;survival\left(\%\right)=\frac{A_{s}-A_{b}}{A_{c}-A_{b}}\times 100\%$$

Where, *A*_*s*_ is the absorbance of experimental cells, *A*_*c*_ is the absorbance of control group cells. And *A*_*b*_ is the absorbance of blank group cells.

### 2.10 Cell migration assay

The cells were digested and incubated in 12-well plate. After the cells are filled with each septal hole, the 1 mL gun head was perpendicular to the hole plate to make cell scratches, and the width of each scratch was ensured as far as possible. The cell culture solution was removed, and the orifice plate was rinsed with PBS three times to wash away the cell debris generated by scratches. Add the hydrogel soak solution and culture medium. The culture plates were cultured in an incubator at 37°C, and photographs were taken with an inverted microscope after incubation for 0 h, 12 h, 24 h, and 36 h.

$$Migration\;Rate\left(\%\right)=\frac{S_{1}}{S_{0}}\times 100\%$$

Where, *S*_*0*_ is the scratch area on 0 h and *S*_*1*_ is the scratch area on different time.

### 2.11 *In vitro* inflammation test

Mouse mononuclear macrophages (RAW264.7) were inoculated in 96-well plates, LPS (5 μg/mL) was used to induce inflammation in the model for 24 h, and EPL-TBA-40 hydrogel of different concentration was added to the 96-well plates, and co-incubated for 24 h. The levels of NO, IL-6, and TNF-αin the supernatant were determined by the Griess reaction and ELISA kit.

### 2.12 EPL-TBA hydrogel hemolysis rate test

The hemocompatibility test was based on a method published by (Zou et al., 2022). Briefly, the hemolytic activity of hydrogels was measured as follows. First, EPL-TBA hydrogel was added to a glass bottle and 4% of human red blood cells were added to the surface of the hydrogel. After incubating with the hydrogel for 1 hour, the suspension was centrifuged at 2,500 r/min for 3 min. After photographing the tubes, the supernatant was collected. Microplate reader was used to determine the optical density at 490 nm (OD_490nm_) of the supernatant. The percentage of hemolysis was calculated using the Eq.

$$Hemolysis\left(\%\right)=\frac{A_{0}-A_{1}}{A_{2}-A_{1}}\times 100\%$$

Where, *A*_*0*_ is the absorbance of experimental, *A*_*1*_ is the absorbance of negative control and *A*_*2*_ is the absorbance of positive control.

### 2.13 Normal wound healing study

The wound healing assay was modified from (Xu, et al. 2019). Female Sprague-Dawley (SD) rats (weighing 200–250 g each) were used for constructing the model for a full-thickness skin wound that was performed on rats. The rats were divided into control group and EPL-TBA-40 group with four in each group. The Ethics Council at Zunyi Medical University authorized all animal testing (ZMU23-2,302-009). After anesthetizing healthy mice, full-layer skin wounds (1.0 cm × 1.0 cm) were made on the back of each rat. To keep the hydrogel samples in place, the lesion was treated with EPL-TBA-40 hydrogel before being bandaged with PU film. Using a smart phone, photograph wound healing on days 1, 3, 7, and 10. Image J software was used to measure the trauma area of each group. The percentage of wound area closure was calculated using the Eq.

$$Wound\;area\;closure\left(\%\right)=\frac{S_{1}}{S_{0}}$$

Where, *S*_*0*_ is the wound area on day 0, and *S*_*1*_ is the wound area on different days.

### 2.14 Histological analysis

Wound site tissues were collected at days 3, 7, and 10, fixed with 10% paraformaldehyde solution and embedded in paraffin wax. Tissue sections were stained using hematoxylin and eosin (H&E) staining.

### 2.15 Statistical analysis

All data are expressed as mean ± standard deviation. Two-way analysis of variance (ANOVA) with the Geisser-Greenhouse correction and Tukey’s multiple comparisons test, one-way ANOVA and Holm-Sidak’s multiple comparisons test were used to calculate the significance of differences between groups under test conditions (GraphPad Prism 8.4.0 software, United States).

## 3 Results

### 3.1 Synthesis and characterization of EPL-TBA hydrogel

**FIGURE 1:**
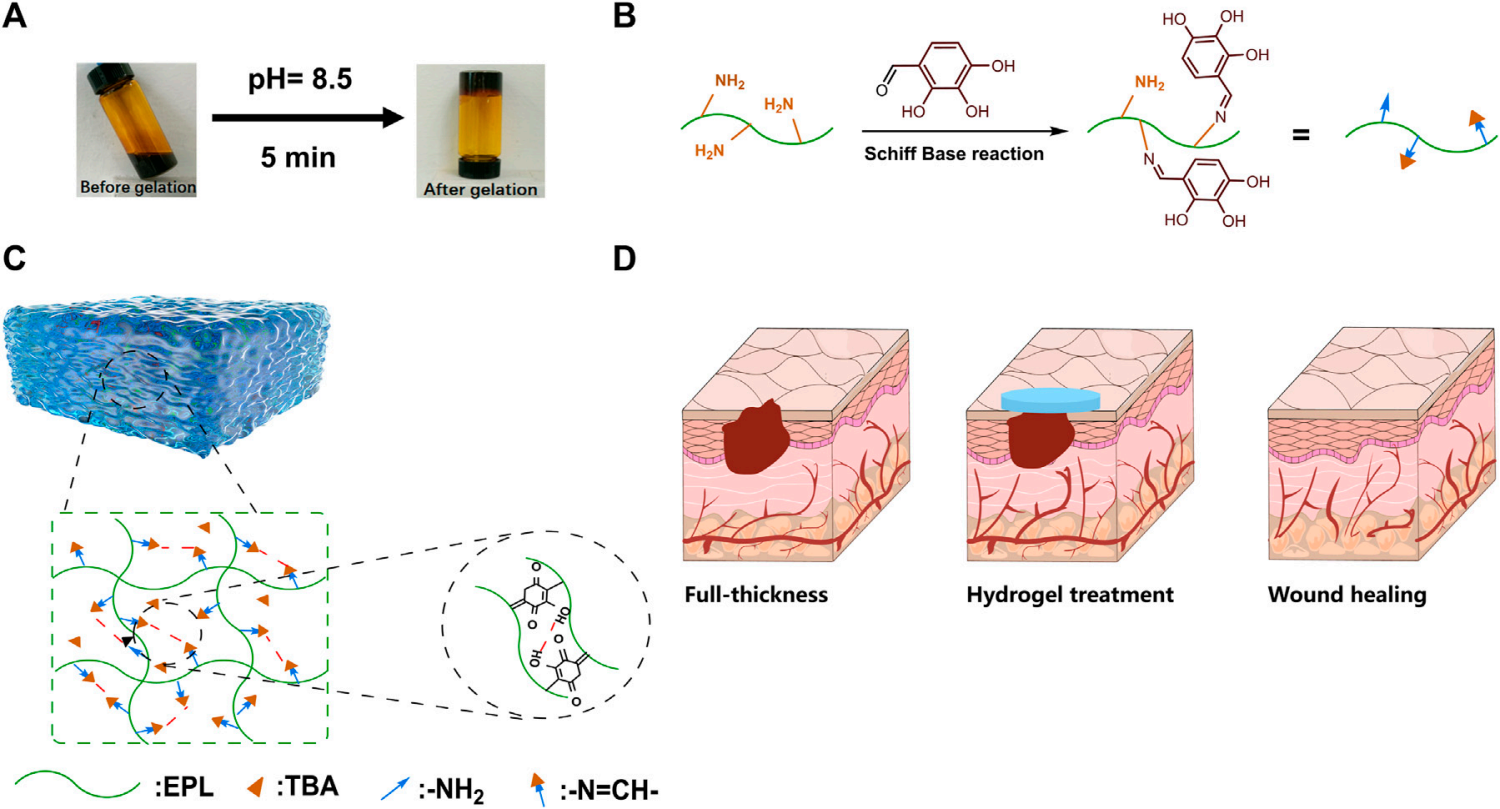
**(A)** EPL-TBA hydrogel vial inverted test. **(B)** Schiff base reaction between amino groups in EPL and aldehyde groups in TBA. **(C)** Schematic diagram of EPL-TBA hydrogel structure. **(D)** EPL-TBA hydrogel for wound treatment.

**FIGURE 2:**
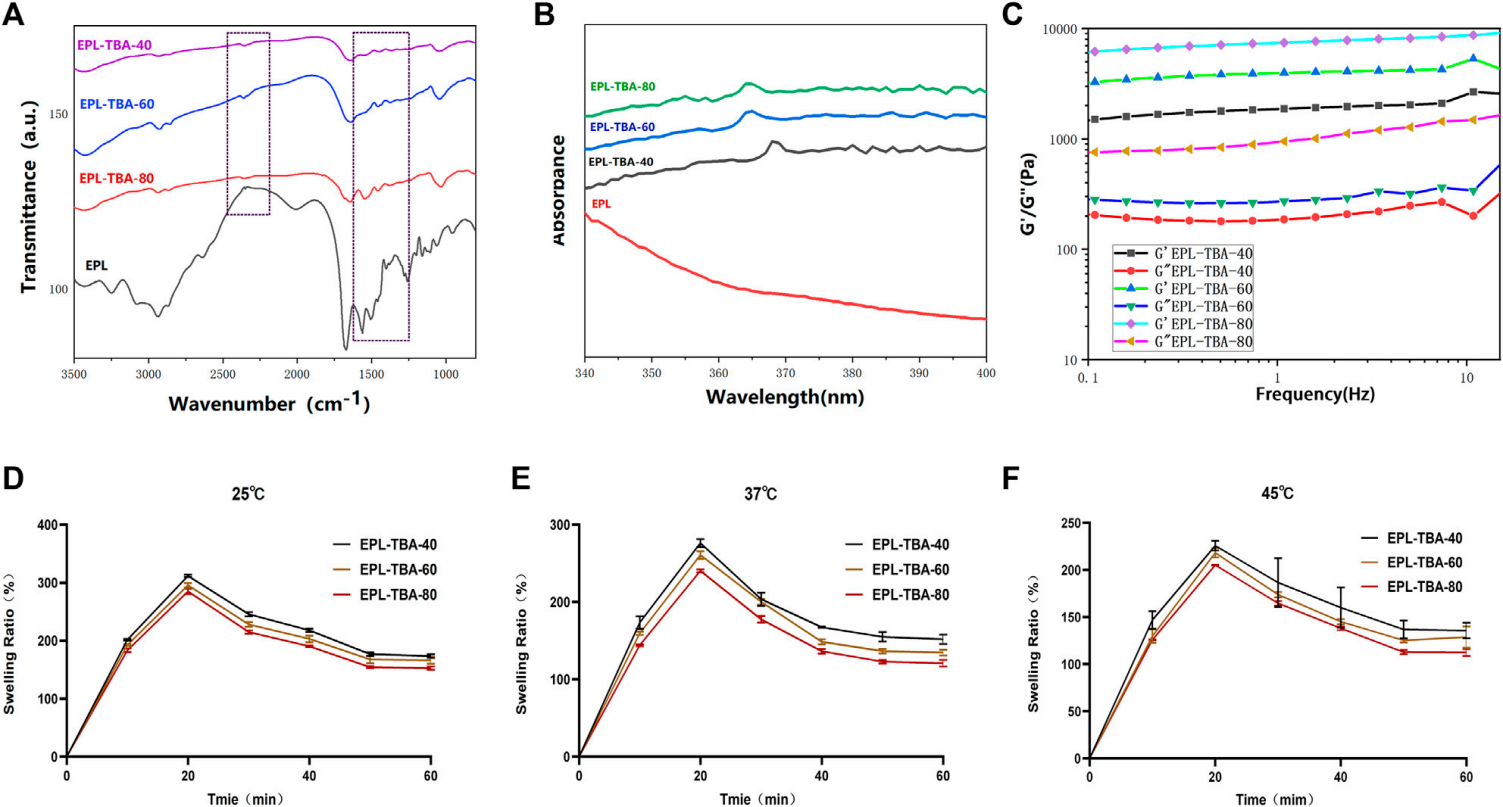
**(A)** FTIR characterization of EPL-TBA hydrogel. **(B)** UV−vis spectra of EPL, EPL-TBA-40, EPL-TBA-60 and EPL-TBA-80. **(C)** EPL-TBA hydrogel frequency scanning test. **(D–F)** Swelling behavior of EPL-TBA hydrogel at different temperature.

The vial inversion experiment demonstrated that the EPL-TBA based visible hydrogel was formed with reaching steady state within 5 min (Figure 1A). EPL-TBA hydrogel is formed by cross-linking the amino group on EPL with the aldehyde group on TBA by the Schiff base reaction (Figure 1B). The schematic structure of the EPL-TBA hydrogel is shown in Figure 1C EPL’s concentration was set at 95 mM, and the levels of TBA (247 mM, 371 mM and 494 mM) were changed to control the amount of cross-linking. The chemical ingredients of EPL-TBA hydrogels, which were determined by FT-IR (Figure 2A). The amide I and II bands, which fell within the *β*-sheet conformation region (Maeda et al., 2003), were assigned the evident characteristic peaks of 1,654 and 1,532 cm^−1^ in EPL, indicating that EPL backbones were already present in EPL-TBA hydrogels. EPL-TBA hydrogels show a peak at 2,452 cm^−1^ due to the typical absorption of freshly synthesized Schiff bases by the amine groups of EPL and TBA, demonstrating that the hydrogel network was formed successfully (Khan et al., 2022). Moreover, compared to EPL, EPL-TBA hydrogels displayed a distinct absorption peak at 350 nm in the UV-vis spectroscopic analysis, demonstrating that the new structure was generated (Figure 2B). Rheological tests were performed to demonstrate the structural stability of EPL-TBA hydrogels (Figure 2C). The reserve modulus (G′) of the EPL-TBA hydrogel remained higher than its loss modulus (G″) over the entire strain range, indicating the gel-like behavior of the sample (Zhang F. et al., 2018). As shown in Supplementary Figure S1A–C, with the increase of shear frequency, the curves of G′ and G″ do not intersect in the strain range, indicating that the structure of hydrogel network will not be destroyed under high frequency shear conditions (Ren et al., 2019). Rheological tests demonstrated that the EPL-TBA gels have excellent structural stability even under high frequency shear conditions.

### 3.2 Analysis of physical properties of hydrogel

The liquid absorption ability of the hydrogels was tested by placing the EPL-TBA hydrogels into PBS solutions at 25°C, 37°C and 45°C. As shown in Figures 2D–F, within 1 h of soaking in PBS, all three EPL-TBA hydrogels exhibited rapid liquid absorption and swelling, with the EPL-TBA-40 hydrogel exhibiting more pronounced liquid absorption than the other two EPL-TBA hydrogels. At 25°C, the EPL-TBA-40 hydrogel reached a high swelling rate of 313.95%. The expansion rates of the other two hydrogels were 295.56% and 285.10%, respectively. The swelling rates of the three hydrogels at 25°C, 37°C and 45°C were not significantly different. EPL contains abundant hydrophilic amino groups, which is the main reason for the superior swelling properties of EPL-TBA hydrogels (Xu, et al., 2019). Since EPL-TBA-40 hydrogel has the highest content of unreacted EPL, it has the highest swelling rates. In addition to the swelling behavior of the hydrogel from the test, the moisturizing properties were also studied. The excellent moisturizing property of the hydrogel could maintain a moist microenvironment at the wound site for more time, which contributes to the formation of wound epithelialization (Santhini et al., 2022). As shown in Supplementary Figure S2, the three EPL-TBA hydrogels were still able to ensure more than 90% of the liquid, which was not lost after being placed at 37°C for 48 h. The EPL-TBA hydrogel can effectively prevent water evaporation and can always keep the EPL-TBA hydrogels in a moist state, therefore accelerating wound healing.

### 3.3 Antimicrobial assay

**FIGURE 3:**
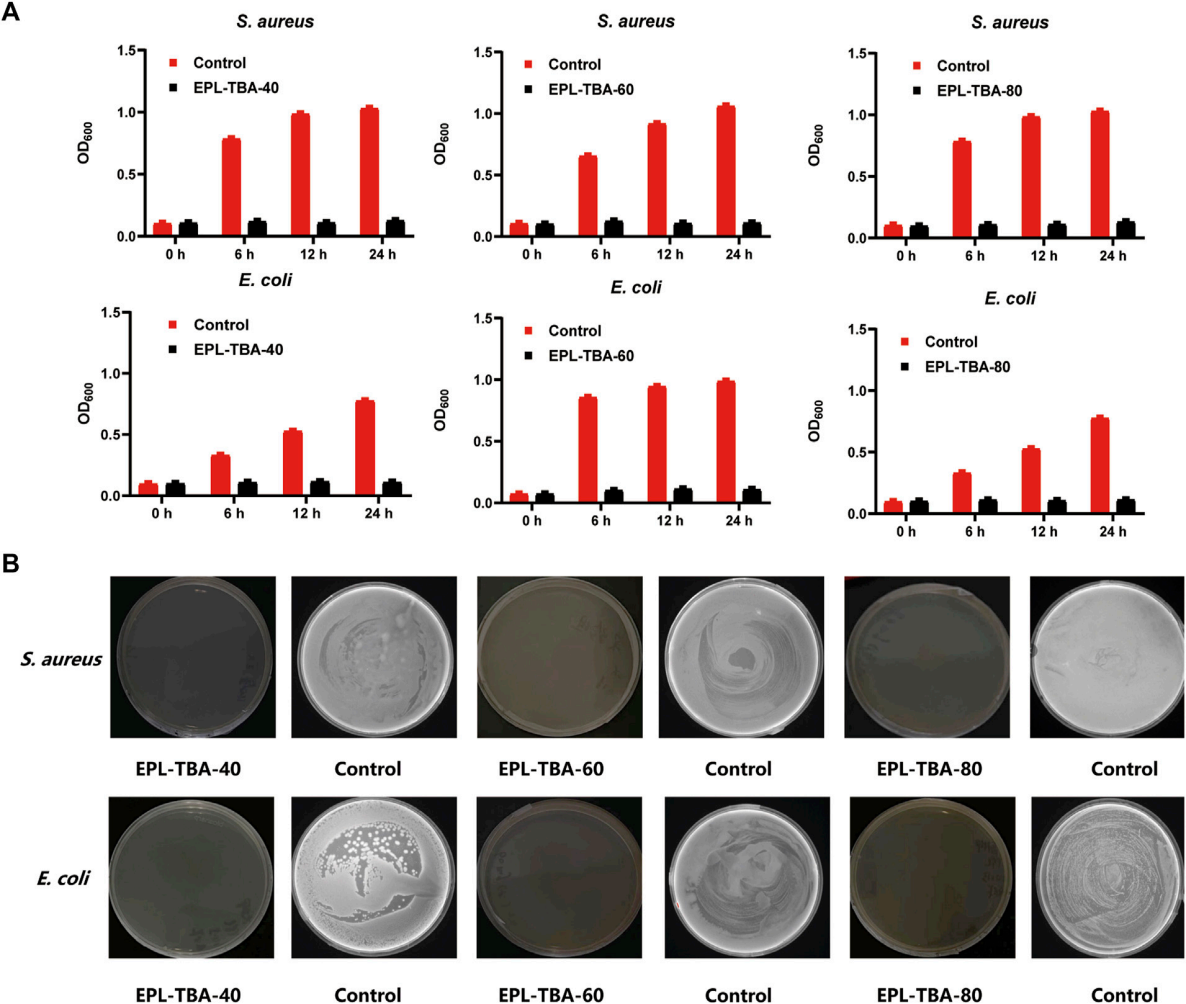
**(A)** Antimicrobial activity of EPL-TBA hydrogel against *S. aureus* and *E. coli* after 0, 6, 12, and 24 h incubation. **(B)** Colony development of *S. aureus* and *E. coli* from bacteria suspensions after 24 h incubation with EPL-TBA hydrogel. As a control, solutions containing neither EPL-TBA hydrogel were applied.

The antimicrobial activity is one of the most important properties of hydrogels used in wound dressings (Ghobril and Grinstaff, 2015). In addition to the ability of a wound dressing to resist external bacteria from entering its own wound tissue, an ideal wound healing dressing with effective antimicrobial properties would be more attractive (Zhao et al., 2017; Alves et al., 2020). Firstly, we tested the minimum inhibitory concentrations (MIC) of EPL and TBA, and the results showed that MIC of EPL for both *S. aureus* and *E. coli* was 4 μg/mL, and MIC of TBA for both *S. aureus* and *E. coli* was 30 μg/mL (Supplementary Table S1). In order to characterize the antibacterial ability of hydrogels, the Gram-positive bacteria *S. aureus* and *MRSA*, Gram-negative bacteria *E. coli* and *P. aeruginosa* were selected as experimental strains in our study. After 24 h incubation demonstrated that the EPL-TBA hydrogel experiment group had nearly no change in OD_600nm_, while the control group *S. aureus* and *E. coli* gradually increase (Figure 3A). Meanwhile, *S. aureus* and *E. coli* treated with EPL-TBA hydrogel did not form bacterial colonies on the agar plates, while the control agar plates were filled with colonies, as shown in Figure 3B. Furthermore, the EPL-TBA hydrogel also inhibited *MRSA* and *P. aeruginosa*, and the absorbance of the bacteria in the experimental group remained basically unchanged, while that of the blank group gradually increased (Supplementary Figure S3A). After verification by agar plate coating, no colonies were formed on the experimental group, while the blank group was filled with colonies (Supplementary Figure S3B). These findings demonstrate that EPL-TBA hydrogel can inhibit various kinds of bacteria.

**FIGURE 4:**
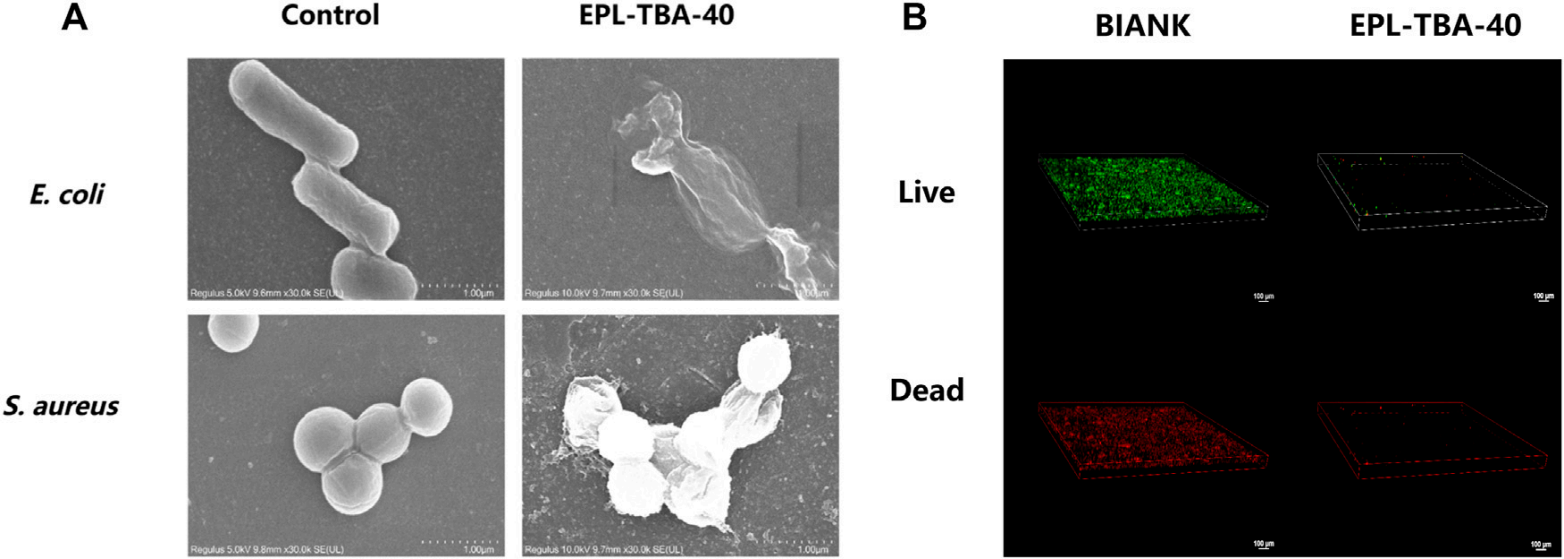
**(A)** SEM images of *E. coli* and *MRSA* cells on the surfaces of control (left) and EPL-TBA-40 hydrogel (right). **(B)** After 24 h of incubation, confocal microscopy images indicate the growth of bacteria on the surface of the blank control (left) and the EPL-TBA-40 hydrogel surface (right). The LIVE/DEAD Backlight bacterial viability kit was used to stain bacteria.

The bacteriostatic effect of cationic peptide hydrogels is achieved by disrupting the cell membranes of anionic bacteria through electrostatic action (Li et al., 2011; Yan, et al., 2021). Scanning Electron Microscope (SEM) was used to observe the morphology characteristics of bacteria in contact with the EPL-TBA hydrogel in order to further investigate the mechanism of killing bacteria. The morphology characteristics of bacteria changed significantly after contact with the EPL-TBA-40 hydrogel, when compared to control bacteria with intact cells (Figure 4A). The cell membranes of bacteria that had been incubated with hydrogels differed from the smooth cell membranes of intact bacteria (Figure 4A), and the cell membrane surfaces of *E. coli* and *MRSA* were collapsed or even broken (Figure 4A). These results demonstrated that the cell membrane of EPL-TBA incubated bacteria underwent collapse and breakage, which in turn led to the death of the bacteria.

### 3.4 Biological film assay

The bacterial biofilms encapsulate the bacterial body in layers, delaying or inhibiting the penetration of antimicrobial drugs and compromising the antimicrobial effect, leading to further difficulties in wound healing (Sharma et al., 2016; Johani et al., 2017). Inhibiting the biofilm formation remains a key strategy to prevent wound infection. In our study, *MRSA* was cultured using EPL-TBA-40 hydrogel and control quartz slides to form biofilms. Bacterial biofilm formation was observed using confocal microscopy and LIVE/DEAD staining. Biofilm formation diagram using quartz slide incubation (Figure 4B). A biofilm formed when a lot of living bacteria and several dead bacteria adhered to the quartz plate. Nevertheless, on the surface of the EPL-TBA-40 hydrogel, no bacterial biofilms were observed. (Figure 4B). This occurrence is explained by the fact that when bacteria come into contact with EPL-TBA hydrogel, they are already killed by the EPL-TBA hydrogel and cannot form a biofilm, thus preventing the formation of a biofilm. EPL-TBA hydrogel can remove the biofilm and prevent the production of drug-resistant bacteria.

### 3.5 Biocompatibility of EPL-TBA hydrogel

**FIGURE 5:**
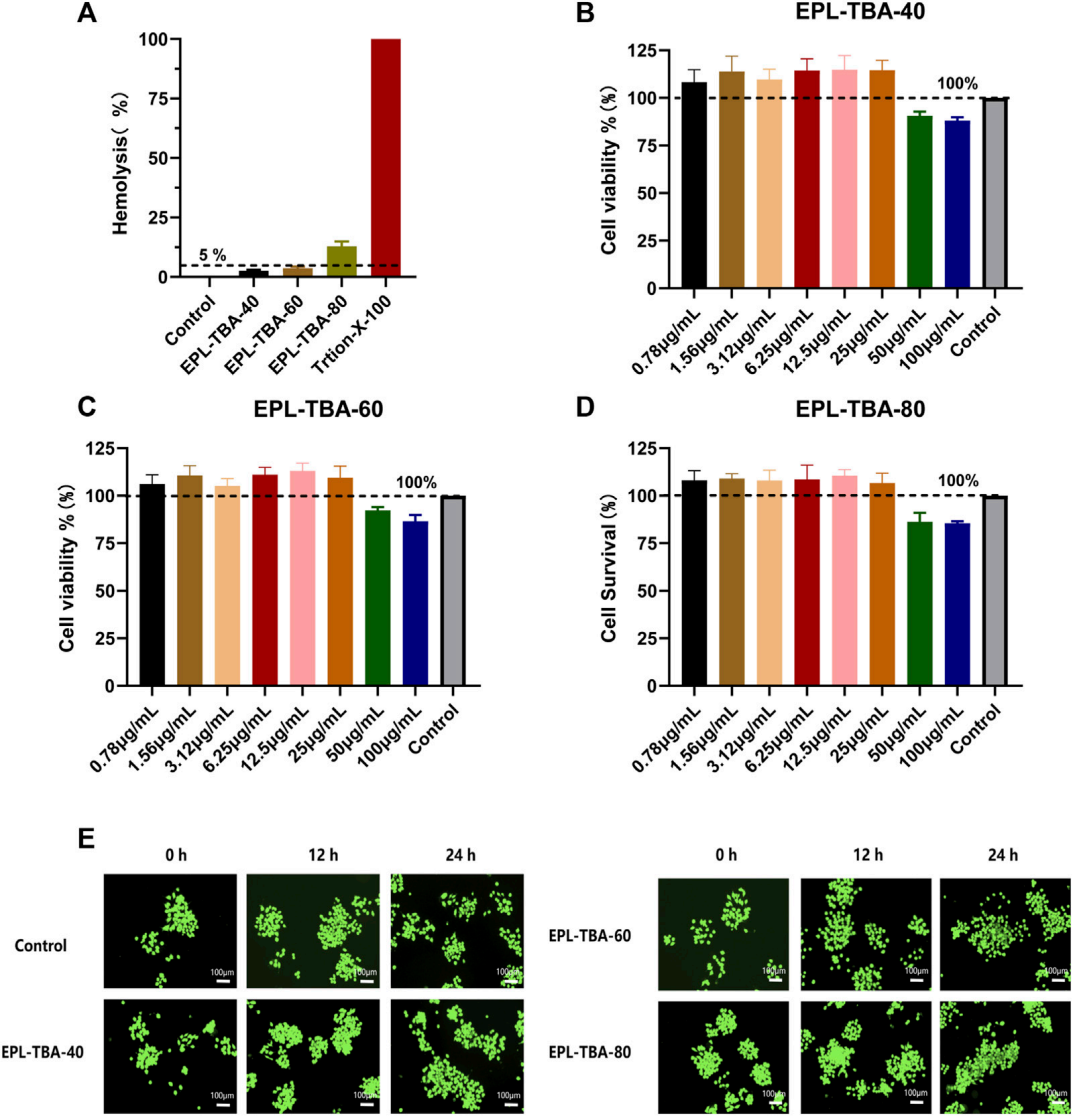
**(A)** Hemolysis assay of EPL-TBA hydrogel using human erythrocyte. **(B–D)** The effect of EPL-TBA-40, EPL-TBA-60 and EPL-TBA-80 on BNCC337722 cells via CCK-8 assay. **(E)** The LIVE/DEAD Backlight cell viability kit was used to stain BNCC337722 cells. Scale bar = 100 μm.

The hemolytic activity of EPL-TBA hydrogels on human erythrocytes and their cytotoxicity to BNCC337722 cells were determined. Hemolysis may occur when erythrocytes rupture after direct contact with different hydrogels in blood. The hemolysis assay evaluates the degree of *in vitro* hemolysis of the material by measuring the amount of hemoglobin. The average hemolysis rates of EPL-TBA-40, EPL-TBA-60, and EPL-TBA-80 for red blood cells are 2.61%, 3.73%, and 12.93%. Respectively, as shown in Figure 5A, indicating that EPL-TBA-40 and EPL-TBA-60 have excellent biocompatibility for red blood cells. The toxicity of EPL-TBA hydrogel to cells is one of the key factors in determining whether the hydrogel can be used as a wound dressing. Furthermore, we carefully investigated the toxicity of different concentrations of EPL-TBA hydrogels on BNCC337722 cells by CCK-8 assay (Figures 5B–D). These findings indicated that different concentrations of EPL-TBA hydrogels did not exhibit toxicity to BNCC337722 cells below the concentration of 25 μg/mL. Different concentrations of EPL-TBA hydrogels at 50–100 μg/mL showed slight cytotoxicity, but the cell survival rate was still higher than 80%. BNCC337722 cells were co-cultured with EPL-TBA hydrogel for 0 h, 12 h, and 24 h and stained with LIVE/DEAD Backlight Cell Viability Kit. BNCC337722 cells incubated with EPL-TBA hydrogel had complete morphology and a clear structure. After 36 h of incubation, the number of BNCC337722 cells increased, indicating that EPL-TBA hydrogel has excellent biocompatibility and can promote cell proliferation (Figure 5E). These findings indicated that EPL-TBA hydrogel has excellent biocompatibility and is likely to be a wound dressing.

### 3.6 Cell migration and anti-inflammatory tests

**FIGURE 6:**
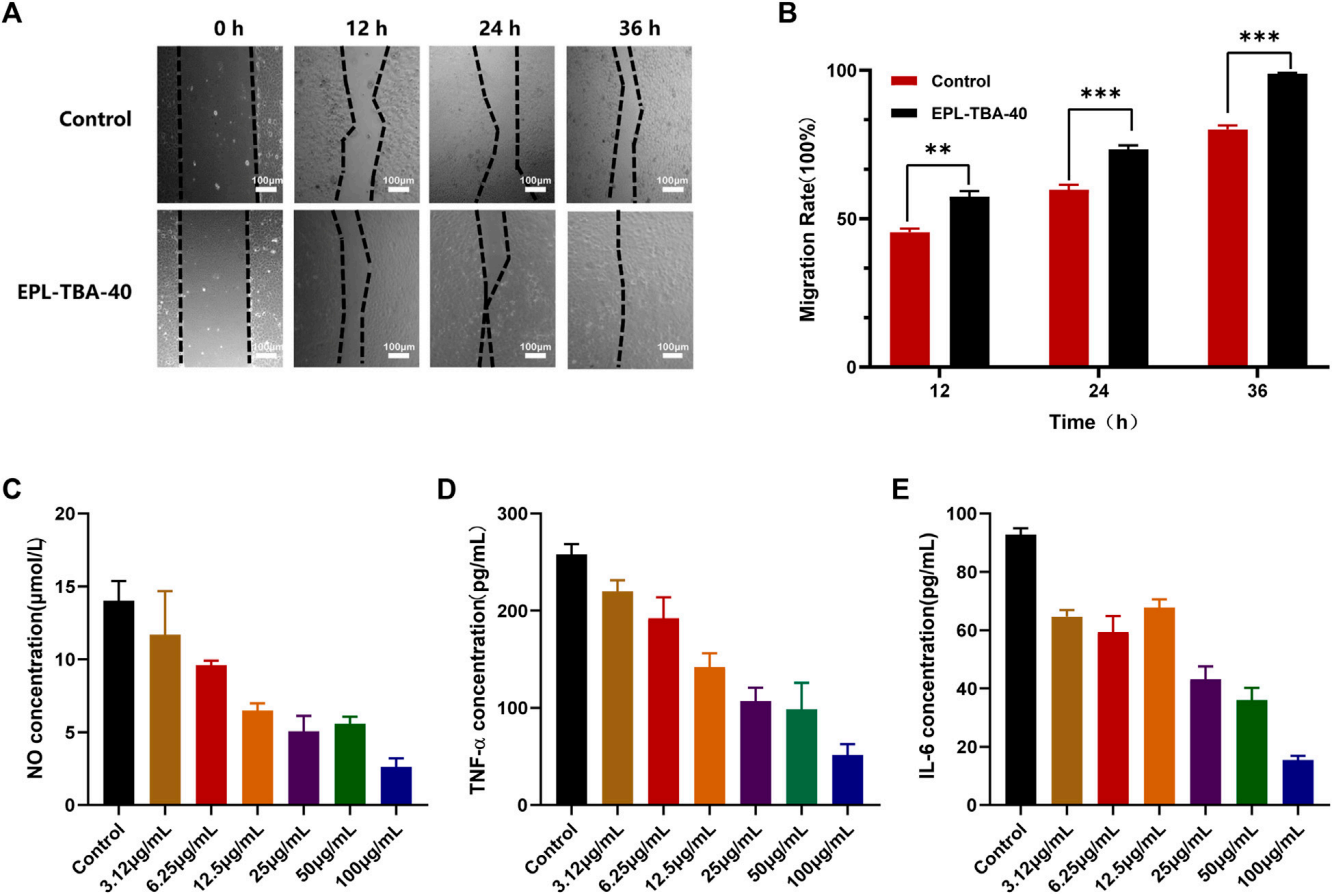
**(A,B)** Mobility of BNCC337722 cells treated with EPL-TBA-40 hydrogel. Scale bar = 100 μm (*p < 0.05, **p < 0.01, ***p < 0.001) **(C–E)** Effects of EPL-TBA-40 hydrogel on NO, IL-6 and TNF-α secreted by LPS-stimulated RAW264.7 cells.

In the cell migration experiment (Figures 6A, B), BNCC337722 cells gradually migrated to the blank area after 36 h of incubation. Compared with the control group, EPL-TBA-40 hydrogel can significantly promote cell migration. The cell mobility of EPL-TBA-40 (98.9%) was higher than that of cells completely culture-induced (80.02%). Furthermore, the anti-inflammatory effect of EPL-TBA-40 hydrogel was investigated by inducing inflammation in RAW264.7 cells by LPS. Compared with the blank group, different concentrations of EPL-TBA-40 hydrogel could significantly reduce the concentrations of NO, IL-6, and TNF-α in a concentration dependent manner (Figures 6C–E). These findings demonstrate that EPL-TBA hydrogels promote cell migration while also inhibiting the production of inflammatory cytokines, which would be benefit for wound healing.

### 3.7 Normal wound healing effect

**FIGURE 7:**
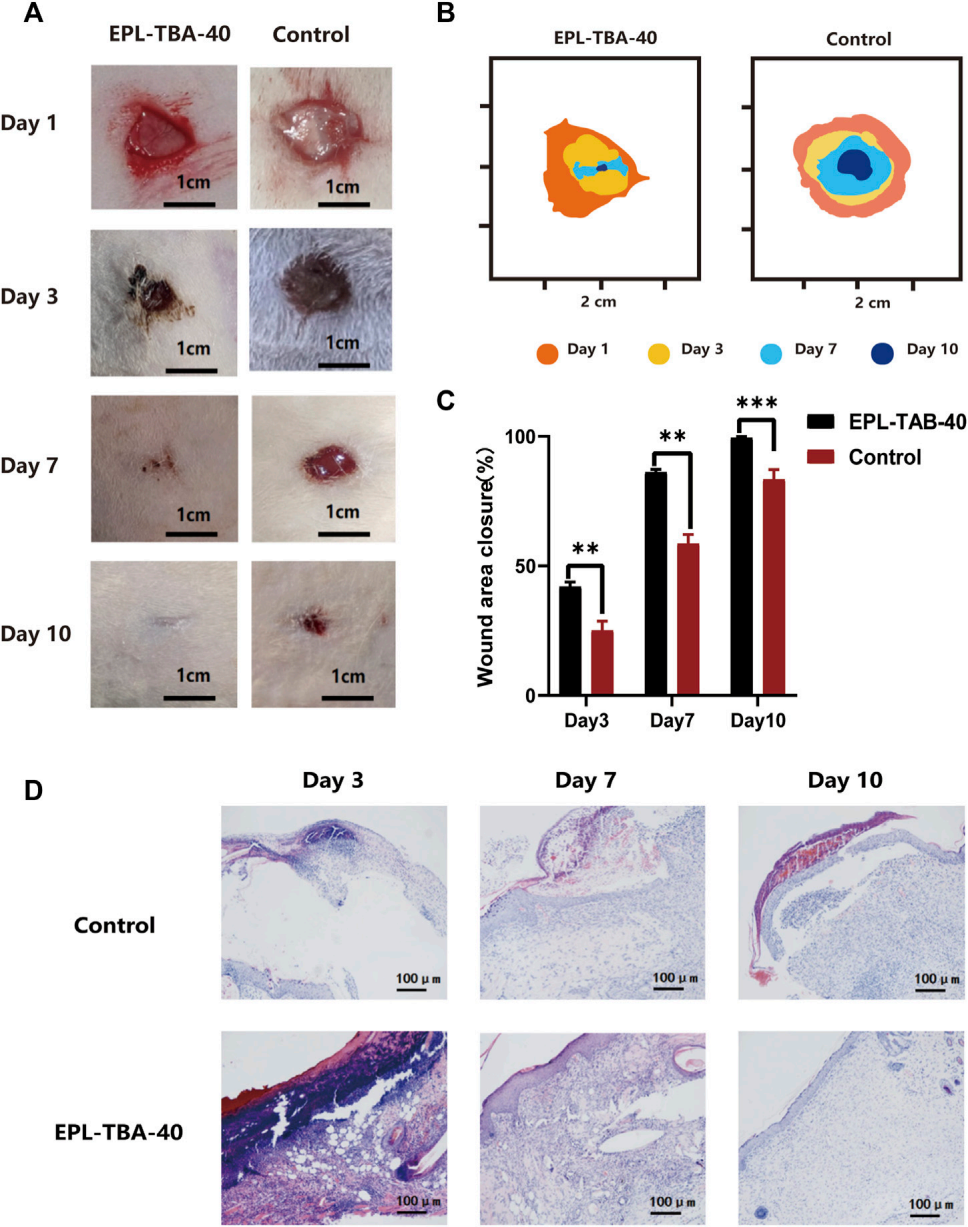
**(A)** Wounds of untreated wound group and EPL-TBA-40 hydrogel treatment group on day 1, 3, 7, and 10. (scale bar: 1 cm). **(B)** Schematic diagrams of contraction changes on days 1, 3, 7 and 10 during wound healing. **(C)** Schematic diagram of the wound area (*n* = 4, **p < 0.01, ***p < 0.001). **(D)** Histological analysis was performed on blank group and EPL-TBA hydrogel group on day 3, day 7 and day 10 (*n* = 3).

To assess the therapeutic effect of EPL-TBA hydrogel on the entire skin normal wound, rats were divided into two groups, a blank control group and EPL-TBA-40 hydrogel as an experimental group, and the hydrogel was changed at 1st, 3rd, 7th, and 10th days after surgery. We tracked and statistically analyzed wound closure markers using smart photographs of the wound and quantitative displays of closure rate (Figures 7A, B). As shown in Figure 7A, depicts the physical map of wound healing at various time periods, and it is obvious that the wound healing rate of the EPL-TBA hydrogel group was much faster than the other groups throughout the treatment period. The initial wound size for both groups was about the same at day 0. On day 3, the blank control wounds did not reduce appreciably, whereas the wounds in the EPL-TBA-40 hydrogel group all crusted and shrank. On day 7, the blank group began to show significant wound contraction, and the hydrogel group basically healed, with better wound healing in the EPL-TBA-40 hydrogel group and wound closure rates 58.64% and 86.21%, respectively. On day 10, the wound closure area of the blank control group increased, a small amount of hair appeared around the wound, and the wound closure rate was only 83.47%, respectively (Figure 7C). In contrast, in the EPL-TBA-40 hydrogel group, wound healing was nearly complete, and new hairs began to cover the wound surface.

### 3.8 Skin histological analysis

Wound healing was assessed by histological study of wound tissue on days 3, 7, and 10. (Figure 7D). H&E staining of the surrounding wound tissue helps better understand the effects of EPL-TBA hydrogel treatment. The wound tissue was infiltrated by abundant of inflammatory cells such as neutrophils, which can be seen in blue in H&E staining. On the third day, both the blank group and the EPL-TBA-40 hydrogel group had some areas of scab, but the EPL-TBA-40 hydrogel group had significantly more scab than the blank group. Additionally, a small amount of capillary regeneration could be seen microscopically in the EPL-TBA-40 hydrogel group. The fact that there were relatively fewer inflammatory cells in the wounds that were coated by EPL-TBA-40 hydrogel, in comparison to the wounds in the blank group, indicating EPL-TBA-40 hydrogel may be able to reduce the inflammation that is present around the wounds. After 7 days, the skin of the blank group did not entirely return and showed signs of perhaps still being necrotic tissue with a small amount of fluid collection, more inflammatory cells and fibroblasts were evident, and hair follicles, glands, and capillaries were apparent. Compared with the blank group, the epidermal layer of the wound covered by EPL-TBA-40 hydrogel had recovered, the epidermal layer was thickened, and inflammatory cells and fibroblasts were visible microscopically, while hair follicles, glands, and capillaries could be observed. After 10 days, the local epidermal layer of the blank group did not recover; crusting and bleeding were visible; the epidermal layer was slightly thickened; and more inflammatory cell infiltration was visible in the dermis. Compared with the blank group, the wound recovered to nearly normal skin; the epidermis was slightly thicker; granules were discharged from the epidermis layer; a small amount of inflammatory cells and fibroblasts were visible in the dermis layer; and hair follicles and glands were visible. On days 7 and 10, the epidermal layer of the EPL-TBA-40 hydrogel group was seen to have recovered or recovered to near-normal skin, with granular discharge visible in the spiny cell layer (consolidation of the epidermal layer) and new hair follicles, glands, and capillaries visible. The recovery effect was significant compared to the blank group. Those findings demonstrate that EPL-TBA hydrogel can promote wound healing.

## 4 Discussion

Here a fast cross-linking hydrogel was reported within only 5 min under mild reaction conditions (Figure 1), and the hydrogels were formed via Schiff base reaction between the amino groups in EPL and the aldehyde groups in TBA (EPL-TBA). The rich catechol groups on the surface of EPL-TBA hydrogel can interact with the surface of various materials through strong hydrogen bonding, hydrophobic, electrostatic, coordination and so on. Since the EPL consists of hydrophilic lysine, which is enriched in amino groups, it can absorb liquid through the interaction between ions to form a hydration layer (Xu, et al., 2019). Therefore, EPL-TBA hydrogel can absorb substantial quantities of liquid (Figure 2). EPL-TBA hydrogel possesses excellent swelling properties that keep wound moist, absorb wound exudate, and promote wound healing (Figure 2). Furthermore, EPL-TBA hydrogel can retain a large amount of liquid within 48 h (Supplementary Figure S2), which can reduce mechanical damage caused by wound dryness and promote wound tissue growth.

*ε*-Poly-_*L*_-lysine (EPL) and 2,3,4-trihydroxybenzaldehyde (TBA) were picked the main as the main components of hydrogel due to their antibacterial effects on Gram-positive and Gram-negative bacteria. Their excellent antimicrobial ability was demonstrated by MIC assay (Supplementary Table S1). Despite the fact that no antimicrobial were biocides released from the EPL-TBA hydrogel, they were still able to kill bacteria through contact with them. All three EPL-TBA hydrogels showed strong antibacterial activity against *S. aureus*, *E. coli*, *MRSA*, and *P. aeruginosa*, indicating that EPL-TBA hydrogels were proven to have broad spectrum antibacterial property (Figure 3). The contact active cationic hydrogels can interact with anionic components of bacteria, such as lipopolysaccharide and wall/lipophosphate, resulting in damage to the integrity of bacterial cell membranes (Li, et al., 2011). Thus, the positive charge density of contact cationic hydrogels is closely related to its bacteriostatic effect. The EPL-TBA hydrogel contains a large amount of EPL, resulting in a large positive charge in the hydrogel, which is highly effective in killing negatively charged bacteria. Bacteria incubated with EPL-TBA-40 hydrogel were observed by SEM. *MRSA* and *E. coli* cells collapsed and ruptured after contact with EPL-TBA-40 hydrogel (Figure 4A). EPL-TBA hydrogel can remove bacterial biofilm and prevent the formation of drug-resistant bacteria and chronic wounds (Zhang C. et al., 2018). This is due to the fact that EPL-TBA hydrogel contains abundant EPL, which can interact with negatively charged bacteria electrostatically and destroy their cell walls and cell membranes, resulting in the death of bacteria and thus achieving effective removal of bacterial biofilms (Figure 4B).

In this study, EPL-TBA hydrogel not only had strong antibacterial activity, but also showed excellent biocompatibility (Figure 5). It has been demonstrated that EPL is compatible with mammalian cells (Zhou et al., 2011; Zou et al., 2018; Xu, et al., 2019). Although the phenol and aldehyde in the TBA molecule are slightly toxic to mammalian cells (Su et al., 2010). TBA cytotoxicity will be greatly reduced by the Schiff base reaction between EPL and TBA. Our results showed that EPL-TBA hydrogel has excellent biocompatibility and is expected to be a new dressing to solve the problem of wound healing. The EPL-TBA-40 hydrogel performed better than the other two groups on the swelling behavior test. Meanwhile, in the hemolysis test, the hemolysis rate of EPL-TBA-40 was lower than 5%, while the hemolysis rate of the other two groups of hydrogels was higher than 5%. Although the three groups of hydrogels all showed excellent antibacterial properties, we chose EPL-TBA-40 as the experimental group instead of the other two groups based on the experimental results of swelling behavior test and hemolysis rate.

The researchers found that quinone-rich biomaterials induce endothelial cells to release biogenic factors that promote cell migration (Yang et al., 2012). EPL-TBA-40 hydrogel promoted the migration of BNCC337722 cells (Figure 6A), which might be attributed to the fact that EPL-TBA-40 hydrogel promoted the release of growth factors in BNCC337722 cells and accelerated the migration of cells. Almost all injuries that occur in tissues are accompanied by inflammation. In addition, inflammatory cells produce a large number of cytokines, and the type and concentration of cytokines greatly affect the repair of damaged tissues (Kotas and Medzhitov, 2015; Forrester et al., 2018; van Horssen et al., 2019). EPL-TBA-40 hydrogel can significantly reduce the production of NO, IL-6 and TNF-α by inflammatory cells (Figures 6C–E). These findings confirm that EPL-TBA hydrogels can promote cell migration while reducing the inflammatory cytokines produced by inflammatory cells, thus accelerating wound healing.

Finally, a rat wound model was used to validate the influence of EPL-TBA-40 hydrogel on healing process. Those findings clearly proved that normal wounds treated with EPL-TBA-40 hydrogel healed faster (Figures 7A–C). Histological analysis discovered that EPL-TBA hydrogel-treated wounds had fewer inflammatory cells, causing faster wound healing (Figure 7D).

To sum up, EPL-TBA hydrogels have the following advantages. First, the EPL-TBA hydrogel is easy to synthesize compared to most of the current hydrogel synthesis methods, and the hydrogel can be formed by a simple chemical reaction. Second, the EPL-TBA hydrogel can be prepared within 5 min, and the chemical reagents used are common and cost-effective, which lays the foundation for future commercialization. Third, the EPL-TBA hydrogel exhibits excellent *in vitro* antibacterial properties and also prevent bacterial biofilm production. The biocompatibility of the EPL-TBA hydrogel was further verified, and good biocompatibility was demonstrated for both hemoglobin and human epithelial cells. Finally, by constructing a rat full-thickness wound model, EPL-TBA hydrogel could substantially promote wound healing. In conclusion, EPL-TBA hydrogel is expected to be a commercially available wound dressing because of its significant advantages and its ability to promote wound healing while preventing bacterial infection.

## 5 Conclusion

In this study, we prepared a rapidly formed antimicrobial peptide hydrogel (EPL-TBA) by a simple chemical reaction. The Schiff base reaction between the amino groups in EPL and the aldehyde groups in TBA makes the hydrogel fast cross-link in 5 min. The EPL-TBA hydrogels exhibit excellent physicochemical properties. Meanwhile, a series of antibacterial experiments indicated that EPL-TBA hydrogel possesses broad-spectrum antibacterial capabilities and can remove biofilms. Furthermore, EPL-TBA hydrogel has excellent biocompatibility. EPL-TBA-40 hydrogel promoted cell migration while suppressing levels of inflammatory cytokines. More importantly, the full-thickness wound model showed that EPL-TBA-40 hydrogel not only promotes wound healing but also reduces the production of inflammatory cells to promote wound healing. These studies demonstrate that EPL-TBA hydrogel is expected to be a kind of wound dressing, providing a new idea and method for solving wound healing problems in the future.

## Data Availability

The original contributions presented in the study are included in the article/Supplementary Material, further inquiries can be directed to the corresponding authors.
