# Supplementary material for: A rapid-crosslinking antimicrobial hydrogel with enhanced antibacterial capabilities for improving wound healing
Source: Front Physiol. 2023 May 30;14:1206211. doi: 10.3389/fphys.2023.1206211 (PMC10265121; doi:10.3389/fphys.2023.1206211)

Supplementary Material

A rapid-crosslinking antimicrobial hydrogel with enhanced antibacterial capabilities for improving wound healing

Xi Zhang^12†^, Wanxin Li^1†^, Genying Wei^1^, Yuling Yan^2^, Ruitao He^2^, Yan Wang^1^, Daoyuan Chen^*1^, Xiaofei Qin^*1^

*** Correspondence:** Corresponding Author: qxf2019300426@zmu.edu.cn (X.Q.), chendy25@mail2.sysu.edu.cn (D.C.).

# Table S1. Minimum inhibitory concentration (MIC) of EPL and TBA. The antimicrobial properties of EPL in solution system was determined with two target pathogens.

| MIC(μg/mL) | *E.coli* | *S. aureus* |
| --- | --- | --- |
| EPL | 4 | 4 |
| TBA | 30 | 30 |

# Supplementary Figure 1. (A-C) Linear viscoelastic region of EPL-TBA hydrogel.


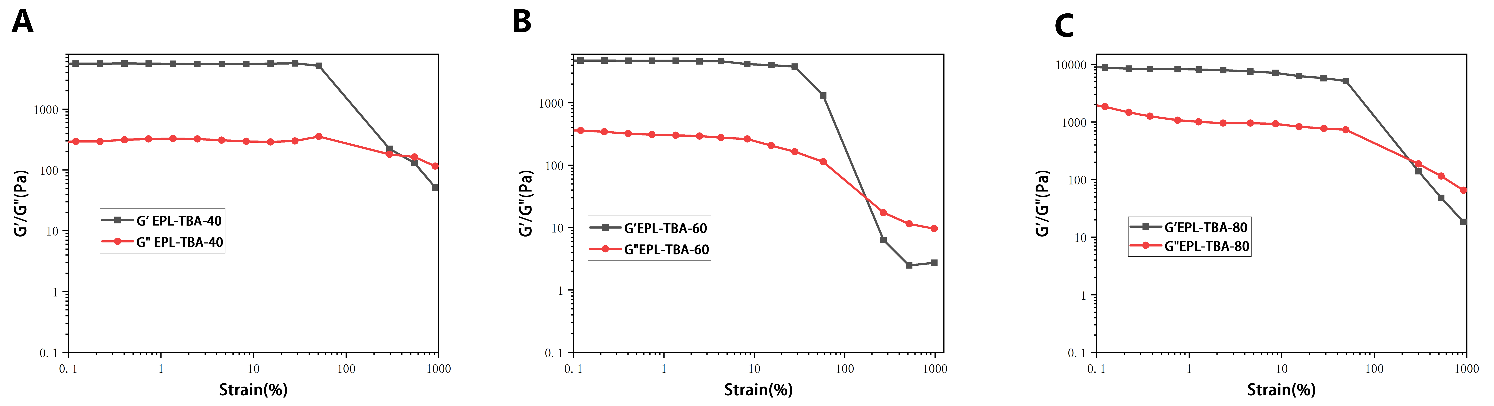


# Supplementary Figure 2. EPL-TBA-hydrogel moisture retention

#
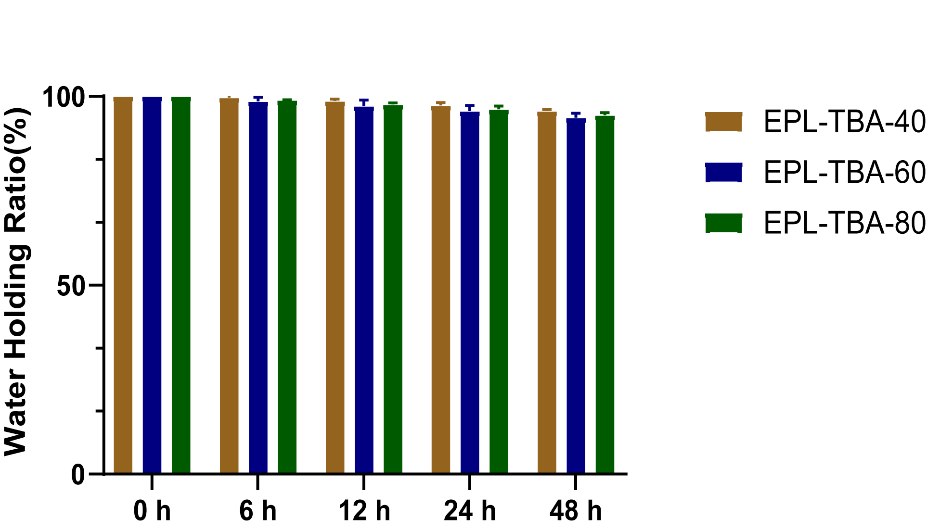


# Supplementary Figure 3. (A) Antimicrobial effect of EPL-TBA hydrogel against *P. aeruginosa*, and *MRSA* after incubation of 0, 6, 12, and 24h. (B) Colony formation of *P. aeruginosa*, and *MRSA* from the suspensions of bacteria after incubation with EPL-TBA hydrogel for 24 h. The suspensions without EPL-TBA hydrogel were used as control.


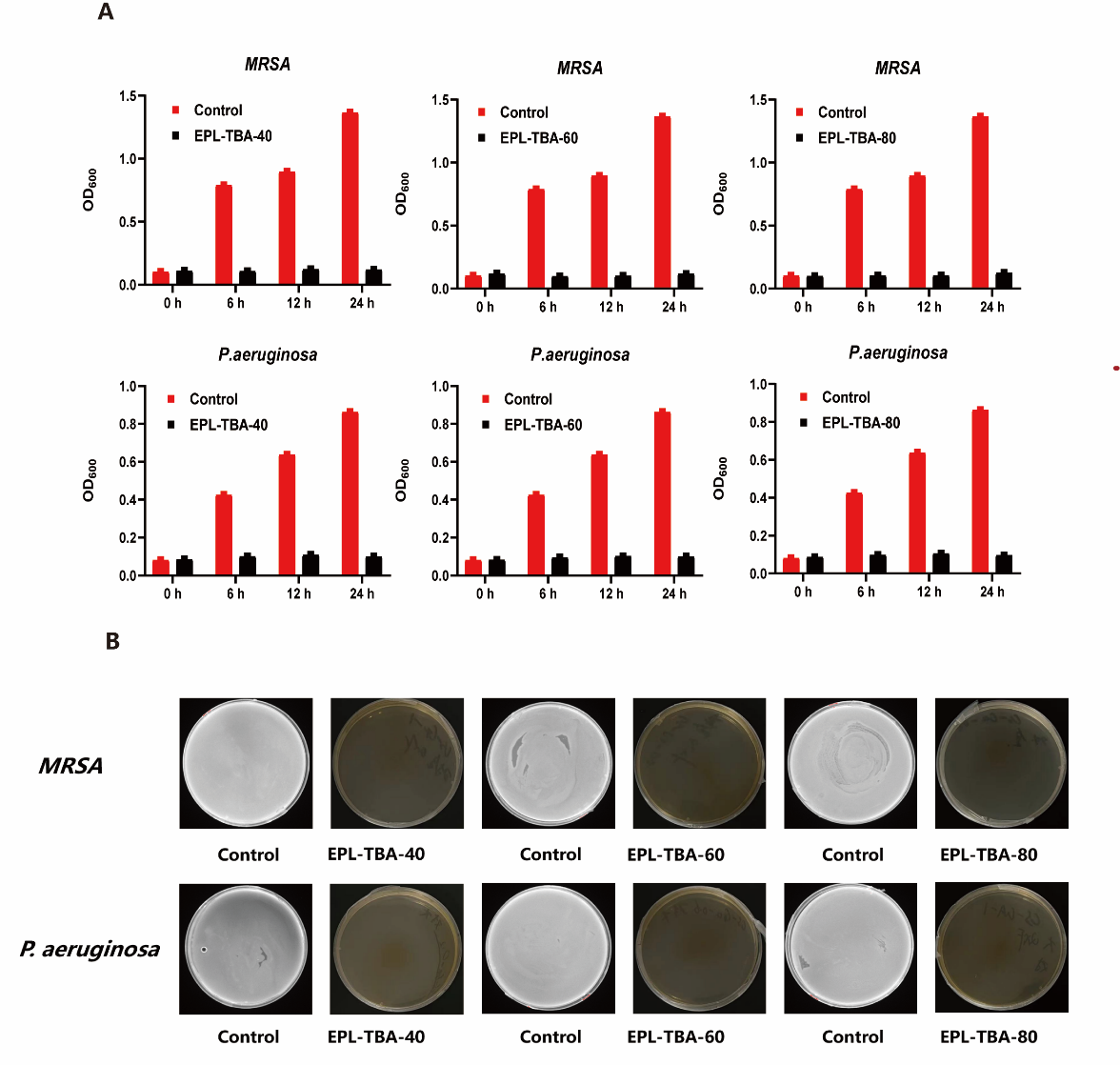

Supplement: Supplementary file 1 [file Table1.docx]
