# Supplementary material for: A rapid-crosslinking antimicrobial hydrogel with enhanced antibacterial capabilities for improving wound healing
Source: Front Physiol. 2023 May 30;14:1206211. doi: 10.3389/fphys.2023.1206211 (PMC10265121; doi:10.3389/fphys.2023.1206211)

Supplementary Material

A rapid-crosslinking antimicrobial hydrogel with enhanced antibacterial capabilities for improving wound healing

Xi Zhang^12†^, Wanxin Li^1†^, Genying Wei^1^, Yuling Yan^2^, Ruitao He^2^, Yan Wang^1^, Daoyuan Chen^*1^, Xiaofei Qin^*1^

*** Correspondence:** Corresponding Author: qxf2019300426@zmu.edu.cn (X.Q.), chendy25@mail2.sysu.edu.cn (D.C.).

# Table S1. Minimum inhibitory concentration (MIC) of EPL and TBA. The antimicrobial properties of EPL in solution system was determined with two target pathogens.

| MIC(μg/mL) | E.coli | S. aureus |
| --- | --- | --- |
| EPL | 4 | 4 |
| TBA | 30 | 30 |

# Supplementary Figure 1. (A-C) Linear viscoelastic region of EPL-TBA hydrogel.


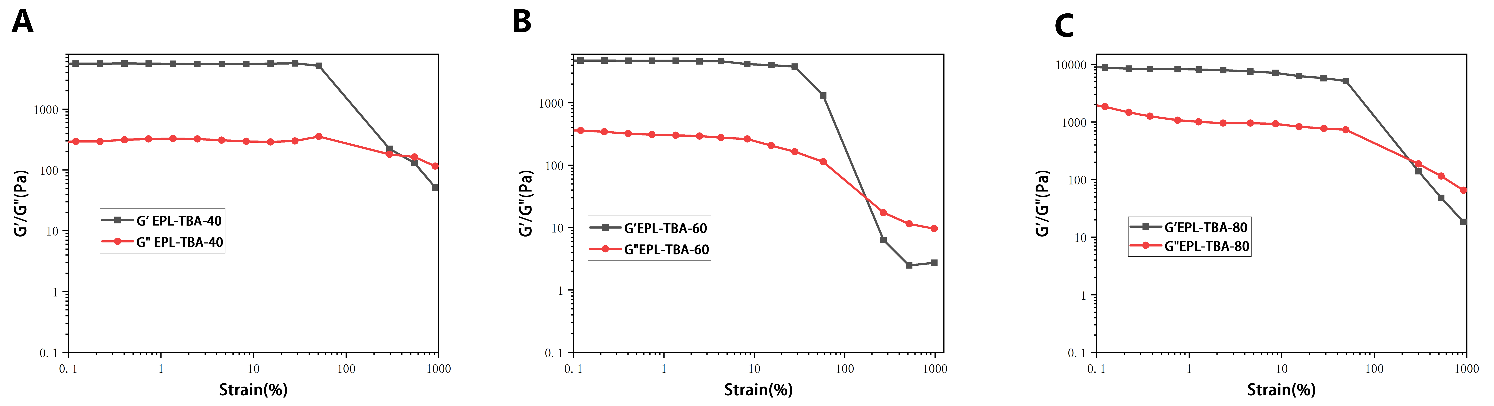


# Supplementary Figure 2. EPL-TBA-hydrogel moisture retention

#
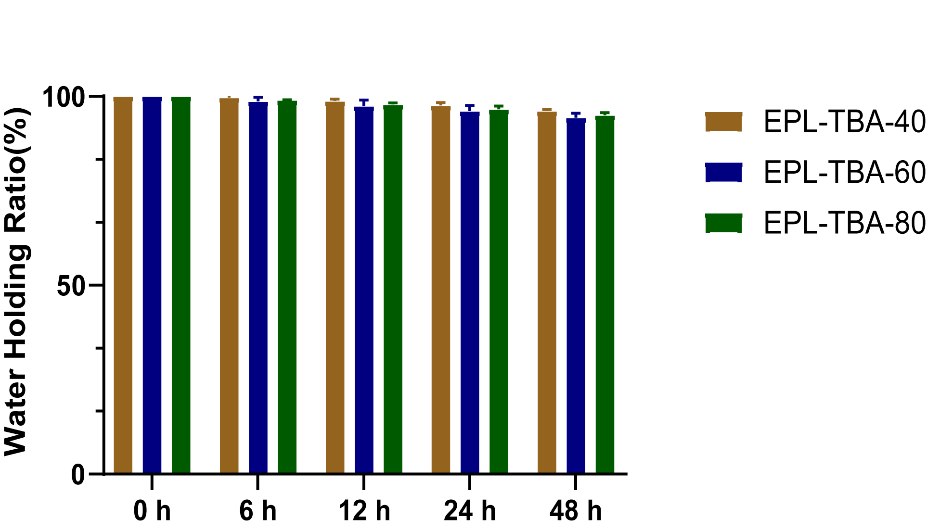


# Supplementary Figure 3. (A) Antimicrobial effect of EPL-TBA hydrogel against P. aeruginosa, and MRSA after incubation of 0, 6, 12, and 24h. (B) Colony formation of P. aeruginosa, and MRSA from the suspensions of bacteria after incubation with EPL-TBA hydrogel for 24 h. The suspensions without EPL-TBA hydrogel were used as control.


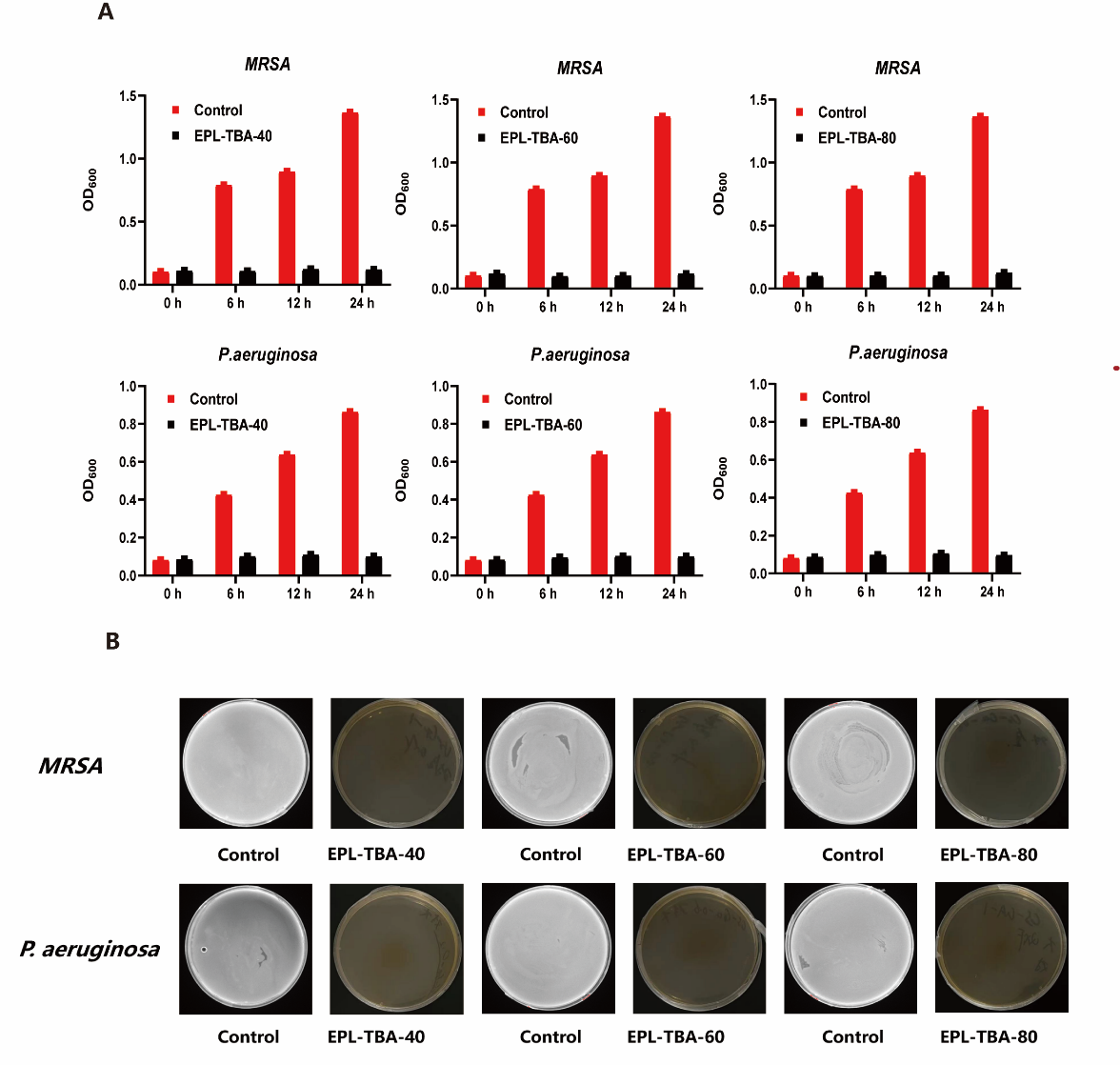

Supplement: Supplementary file 2 [file Table2.DOCX]
